# A Glimpse into Milestones of Insulin Resistance and an Updated Review of Its Management

**DOI:** 10.3390/nu15040921

**Published:** 2023-02-12

**Authors:** Abdullah I. Aedh, Majed S. Alshahrani, Mohammed A. Huneif, Ian F. Pryme, Ramadhan Oruch

**Affiliations:** 1 Department of Internal Medicine, School of Medicine, Najran University, Najran 66324, Saudi Arabia; 2 Department of Obstetrics & Gynecology, School of Medicine, Najran University, Najran 66324, Saudi Arabia; 3 Department of Pediatrics, School of Medicine, Najran University, Najran 66324, Saudi Arabia; 4 Department of Biomedicine, School of Medicine, University of Bergen, 5020 Bergen, Norway; 5 Department of Biochemistry and Molecular Biology, School of Medicine, Najran University, Najran 66324, Saudi Arabia

**Keywords:** type 2 diabetes, gestational diabetes, mitochondrial dysfunction, autoimmunity, gut microbiota, reactive oxygen species

## Abstract

Insulin is the main metabolic regulator of fuel molecules in the diet, such as carbohydrates, lipids, and proteins. It does so by facilitating glucose influx from the circulation into the liver, adipose tissue, and skeletal myocytes. The outcome of which is subjected to glycogenesis in skeletal muscle and lipogenesis in adipose tissue, as well as in the liver. Therefore, insulin has an anabolic action while, on the contrary, hypoinsulinemia promotes the reverse process. Protein breakdown in myocytes is also encountered during the late stages of diabetes mellitus. The balance of the blood glucose level in physiological conditions is maintained by virtue of the interactive functions of insulin and glucagon. In insulin resistance (IR), the balance is disturbed because glucose transporters (GLUTs) of cell membranes fail to respond to this peptide hormone, meaning that glucose molecules cannot be internalized into the cells, the consequence of which is hyperglycemia. To develop the full state of diabetes mellitus, IR should be associated with the impairment of insulin release from beta-cells of the pancreas. Periodic screening of individuals of high risk, such as those with obesity, hypercholesterolemia, and pregnant nulliparous women in antenatal control, is vital, as these are important checkpoints to detect cases of insulin resistance. This is pivotal as IR can be reversed, provided it is detected in its early stages, through healthy dietary habits, regular exercise, and the use of hypoglycemic agents. In this review, we discuss the pathophysiology, etiology, diagnosis, preventive methods, and management of IR in brief.

## 1. Introduction

Non-insulin-dependent diabetes (NIDD), as it was previously known, or type 2 diabetes (T2D), also known as adult-onset diabetes, is a form of diabetes characterized by hyperglycemia occurring over a prolonged period. It results from a relative lack of insulin and insensitivity to the physiological action of this hormone [1]. The typical signs and symptoms of the syndrome are polydipsia, polyuria, and weight loss for an unclear reason. Other symptoms that may accompany these changes are polyphagia, feeling unwell, and wounds that fail to heal that would normally do so without special medical intervention [2]. The problem is that the symptoms are subclinical in the beginning and often appear slowly. Specifically, they include hyperglycemia and hypertension, as well as an ischemic spectrum of heart diseases, stroke, and microangiopathy. The latter manifests as retinopathy, renal failure, and poor blood circulation in the extremities and their sequelae. A wide spectrum of nerve damage and cognitive impairments are also some of the chronic complications of this syndrome [3,4]. In other cases, the diagnosis reveals sudden consequent complications such as hyperosmolar hyperglycemic or diabetic ketoacidosis states [5]. Many people, however, show no symptoms and are diagnosed at a routine medical check-up or hospital admission for other pathologies [6]. T2D is a serious condition in that it accounts for about 90% of all diabetes cases around the world. This is almost 6% of the global population [6,7,8], as shown by statistics from 2015. Global statistical records of 2018 show a higher percentage—about 1 in 11 adults (90% of these have T2D) [9]. Today, there are 425 million people with diabetes mellitus in the world. By 2045, this figure is expected to increase to over 600 million. Certain ethnic groups, and women more so than men, are at a higher risk of contracting the disease [7,10,11,12]. This might be attributed to sedentary professions, as it often accompanies the hormonal changes of pregnancies. Genetics also has a lot to say regarding etiology [1]. The World Health Organization (WHO) has recognized T2D as a global epidemic [13]. The epidemiological panorama of the condition is continuously changing; previously, it was accepted that it affects the adult population of the globe, but in recent years, it has increasingly affected the pediatric age group (adolescents), hand-in-hand with the rising rates of obesity in this population group [7]. It is worth mentioning that the rate of diabetes is increasing annually, independently of and non-parallel with the rates of increase in the global population. This increase has been attributed in essence to increasing rates of obesity, sedentary life, and the increased aging of the global population [14].

## 2. Metabolic Syndrome

Metabolic syndrome is the congregation of abdominal obesity (visceral obesity), hypertension, hyperglycemia, hyperlipidemia (triglycerides), and low serum high-density lipoprotein (HDL). At least three of these criteria must exist for the diagnosis of this syndrome. Insulin resistance is indeed a component of this syndrome. When hyperglycemia falls below the threshold to diagnose diabetes mellitus, the condition is called prediabetes. Insulin resistance, prediabetes, and metabolic syndrome share a spectrum of an overlapping area, thus they are closely related conditions and are milestones of a spectrum of a huge metabolic disorder of energy utilization and storage. Metabolic syndrome is indeed a serious condition in that it is a potential risk factor for ischemic heart diseases and T2D. It is noteworthy to mention that about 25% of the American adult population suffers from this disease [15]. It is thus a hot subject of ongoing research.

### 2.1. Type 2 Diabetes

Type 2 diabetes is the end stage of the pathologic spectrum of metabolic syndrome, in which hyperlipidemia, low levels of HDL, and hypertension are also prominent features of the syndrome in the Western world [16]. The essential and first millstone of this metabolic disorder is IR. This may be accompanied by a relative reduction in insulin secretion in response to carbohydrate ingestion [6]. This phenomenon has been explained by relying on the hypothesis of improper responsiveness of insulin receptors to the insulin being released postprandially (2 h after eating). This type of diabetes is accepted as the most common subcategory within diabetes mellitus. Things are intermingled in a very complicated image in such a way that many individuals with this disorder demonstrate evidence of the prediabetes state, which is manifested by impaired glucose tolerance and increased fasting blood glucose [17]. To reverse or at least slow down the development cascade of T2D is possible by two important means: (1) changing the lifestyle, which includes adopting healthy nutritional habits and practicing regular bodily exercises, and (2) medications that can reduce glycogenolysis (by the liver) and decrease glucose absorption from the intestine, and thus improve insulin sensitivity [18]. As T2D is an inevitable consequence of neglected cases of IR, the most effective preventive measure health caregivers should consider when managing these individuals is providing strong advice to fight obesity. It is true that T2D has genetic predisposing factors, but environmental factors have a lot to say in this context [19]. Other environmental elements that can ignite diabetes mellitus include stress, sedentary life, bad dietary habits, and urbanization, as has been stated elsewhere in this work [20]. The consumption of trans and saturated fatty acids in addition to sweetened drinks (with sucrose) is among the prominent predisposing factors to developing T2D [21,22,23]. In general, foodstuffs characterized by having a high glycemic index (ranging between 70 and above) should be avoided, such as white rice; see Table 1 [24,25,26,27].

### 2.2. Obesity

Obesity is closely related to IR [28]. A body mass index over 30 is accepted as obesity. Obesity is a consequence of IR [25]; it deteriorates diabetes and makes management more difficult. One thing that should be mentioned here is that individuals with greater waist/hip ratios are also at risk of developing diabetes [6]. Obesity and derangement in lipid metabolism play a key role in the pathogenesis and development of insulin resistance, and subsequently T2D, in such individuals. Obese individuals have an increased mass of adipose tissue, which leads to lipid overflow, which in turn causes subtle inflammation via disturbed cytokines’ and adipokines’ secretion [29].

The key to impeding obesity is regular physical exercise and consumption of foodstuff that causes the lowest possible glycemic load (GL). This parameter is closely related to the glycemic index [27]. Glycemic load estimates the value of hyperglycemia that can ensue postprandial levels with the consumption of different types of foodstuff. Each unit of this parameter is equivalent to one gram of glucose [30]. To indicate and discuss all risk factors that are considered potential to predisposing obesity falls outside the scope of this work.

### 2.3. Gestational Diabetes

This is also considered as a variant of IR in that it involves the combination of the relative inadequacy of the amount of insulin being secreted and the responsiveness of different tissues of pregnant women to this hormone. The condition may disappear after parturition [31]. Obstetricians, therefore, recommend that pregnant women check their blood glucose level starting at around weeks 24–28 of pregnancy (as a part of the antenatal workout). If present, the condition is usually diagnosed in the second to third trimester, being attributed to the hormones that antagonize insulin action [32]. The majority of these cases subside after delivery, but unfortunately, some of these women will suffer from T2D or other forms of glucose intolerance after successive deliveries [31]. Solid statistics document that 70% of women with gestational diabetes are at a high risk of developing diabetes (mainly T2D) later in their lives [33,34]. Therefore, antenatal care should not be neglected among pregnant women, especially in societies of the developing world where physical activity is almost non-existent among women in such populations. One point that must be mentioned here is that there exist special body exercises designed for pregnant women as antenatal care measures in different parts of the prosperous world. There are different pieces of evidence that suggest a link between vitamin D deficiency and gestational diabetes [35,36]. The broad lines of management in such cases include dietary changes and regular blood glucose monitoring, and certain cases may also need insulin therapy [37].

### 2.4. Human Insulin in Brief

Human insulin is a peptide hormone (protein) synthesized by the beta cells of Langerhans islets of the pancreas. It consists of two chains (A and B), cross-linked by disulfide bonds between cysteine residues in the corresponding chains and within the same chain, which give this peptide hormone stability, despite it being a small polypeptide of 51 amino acids. It is an anabolic hormone; to be more precise, it is the main anabolic hormone of the human body [38]. This hormone regulates the metabolism of all fuel material in the human diet, that is, carbohydrates, fats, and proteins. It does so by increasing glucose absorption from the circulation, mainly postprandially, into the hepatocytes, lipocytes, and skeletal myocytes for further metabolism [39]. High insulin levels in the blood inhibit glucose secretion from the liver into the blood circulation. The circulating insulin also promotes the synthesis of macromolecules of cellular components such as proteins in cells of different tissues of the human body and lipogenesis in adipocytes—a sort of anabolic effect of this hormone. The reverse situation is encountered when the level of circulating insulin is low, that is, the catabolic process is promoted, especially when the cells start to consume the reserve pools of fat they contain to start with. This physiological process is attributed to the sensitivity of beta cells to blood glucose levels. Insulin secretion increases as a response to a high blood glucose level, and the opposite is true in cases of hypoglycemia. The blood glucose levels indeed lie within physiological limits (4.0 to 5.9 mmol/L preprandial and less than 7.8 mmol/L postprandial, 2 h after a meal). This balance is regulated by the interplay of insulin and glucagon (an alpha pancreatic cell released hormone). Glucagon stimulates glycogenolysis and gluconeogenesis in the liver. The secretion of these two hormones into the circulation is the most pivotal biochemical mechanism to induce glucose homeostasis [40]. Serum insulin levels are measured either in international units (μIU/mL) or as molar concentration (pmol/L) (where 1 μIU/mL is 6.945 pmol/L) [41]. The typical insulin level in the blood between meals is about 8–11 μIU/mL (57–79 pmol/L) [42]. Postprandial insulin release by the pancreas actually comes in bouts, i.e., it is not linear, but rather oscillates up and down. It is believed that this regular fluctuation is necessary to extract insulin from the blood for degradation, which is vital for the down-regulation of insulin receptors in different target cells [43].

### 2.5. Glucose Transporters

Glucose transporters (GLUTs) are a broad group of intramembrane proteins that facilitate the transport of glucose across the cell membrane. These transporters function as uniporters because, in a concentration-dependent manner, they permit diffusion of glucose into the cytoplasm for different catabolic and/or biosynthetic purposes. The human genome encodes 14 different types of these transporters. They are uniporters because they facilitate the diffusion of a single species of a certain molecule (glucose in this case) at a time, along its concentration gradient. This is a passive process in that there is no need for ATP hydrolysis. They are categorized into three main classes, I, II, and III. Class I includes the following subgroups: GLUT1, GLUT2, GLUT3, GLUT4, and GLUT14 [44], which are the most significant and well characterized of these according to Bell et al. [45]. GLUT1 and GLUT3 are the main transporters of glucose into pancreatic beta cells. These membrane proteins, hand-in-hand with sodium-glucose transporters (SGLTs), facilitate this biological task so that insulin in appropriate amounts is released from these cells into the circulation to reach the target organs [46].

#### 2.5.1. GLUT1

GLUT1 is a uniporter membrane protein that facilitates the transport of glucose molecules across the plasma membrane of human cells [47,48]. It expressed mostly in erythrocytes and endothelial cells (of the blood–brain barrier). It is responsible for basal glucose uptake that is sufficient to maintain cellular respiration (electron transport chain) in all cells. This transporter subtype is also widely found in fetal tissues. Expression of GLUT1 increases as intracellular glucose levels fall, and vice versa. The expression is upregulated in neoplastic cells of many tumor types, and this is sustained as such cells need huge amounts of glucose for anaerobic glycolysis (Embden–Meyerhof–Parnas pathway) to maintain the uncontrolled growth of tumors, especially in their deeper zones, as cells in such zones almost completely rely on the anaerobic variant of glycolysis.

#### 2.5.2. GLUT2

This protein has a bidirectional glucose transport function in that it allows glucose influx and efflux across the cell membrane. It is the major glucose transfer gate between the liver and blood [49]. In addition to hepatocytes, its expression predominates widely in renal tubular tissue [50], the small intestine [51], and the beta cells of pancreatic islets. Such bidirectional glucose transport also exists in the basolateral small intestinal enterocytes. This phenomenon is vital for hepatocytes for both glycolysis and glycogenesis, as well as for gluconeogenesis and the release of glucose into the circulation. The level of the circulating free glucose per se is a prerequisite for the beta pancreatic cells to gauge and control glucose levels of the body in a homeostatic balance. The main characteristic of this protein is that all three fuel monosaccharides—glucose, galactose, and fructose—are transported through these from enterocytes into the portal circulation to reach the liver. Because it has a bidirectional action, GLUT2 is a low-affinity transporter subclass; it transports glucose across the membrane from areas of high concentration to areas of lower concentration.

#### 2.5.3. GLUT3

GLUT3 is a high-affinity subclass; it allows glucose transport to occur in all cases, even when glucose levels are intracellularly low—a phenomenon required for neurons [52], placenta, and fetal cells [53]. This transporter protein has a significantly greater transport capacity than GLUT1 and GLUT4, as well as a higher glucose affinity than other subclasses, namely, GLUT1, GLUT2, and GLUT4 [54].

#### 2.5.4. GLUT4

GLUT4 abbreviates glucose transporter protein type 4; it is also known as solute carrier family 2 facilitated glucose transporter member 4. In humans, it is encoded by the gene *SLC2A4* located in chromosome 17. The significance of this transporter protein was demonstrated by James et al. in 1988 and its action is regulated by insulin [55]. This protein has a significant responsibility in glucose storage; it is expressed mainly in adipocytes and striated myocytes, especially skeletal muscles [56]. Like all other GLUT subclasses, the N (amine) and C (carboxyl) ends of GLUT4 are exposed to the cytoplasm and it has 12 transmembrane alpha segments. It is believed that the primary sequence of amino acids in this protein class is what enables them to transport glucose across the cellular biomembranes in which they are studded [57]. The protein also contains the UBX-domain, where ubiquitin can tether to GLUT4 such that the protein can be sequestrated into vesicles in the absence of insulin [58]. GLUT4, when located at the cell surface, by a facilitated diffusion, permits the entry into the circulation of glucose molecules down its concentration gradient into skeletal myocytes and adipocytes. Once glucose is internalized, it is trapped by the process of phosphorylation by glucokinase (liver) and hexokinase; in other tissues, it is trapped by glucose-6-phosphate to enter the glycolytic cycle; or it is polymerized into glycogen depending on the cell type in which glucose molecules diffuse.

#### 2.5.5. GLUT14

GLUT14 has a similar function to GLUT3, but is expressed mainly in the testis [59].

### 2.6. Insulin as a First Messenger in Signal Transduction Cascade

**Figure 1:**
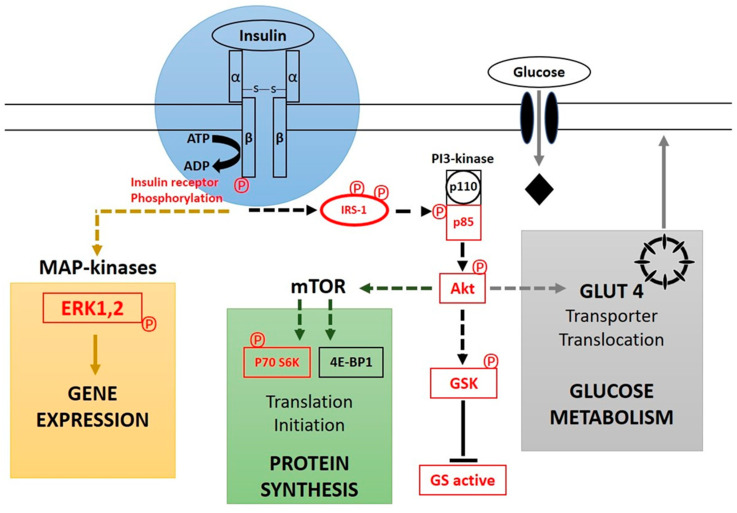
Insulin signal transduction. The insulin receptor (IR) is in blue, the MAP-kinase pathway to gene expression is highlighted in light-brown, and dark-green lines point to the proximal common pathway to protein synthesis (highlighted in green) and glucose metabolism (highlighted in gray). Phosphorylated proteins, IR (insulin receptor), extracellular signal-regulated kinases 1 and 2 (ERK1,2), insulin receptor substrate 1 (IRS-1), the p85 subunit of phosphatidylinositol 3-kinase (PI 3-kinase), Akt protein kinase (Akt), S6 protein kinase (p70 S6K), glycogen synthase kinase (GSK), and glycogen synthase (GS), are in red. P (phosphate group), (–S–S–) disulfide bond, mTOR (mammalian target of rapamycin), ATP (adenosine triphosphate), ADP (adenosine diphosphate) and MAP (mitogen-activated protein).

Insulin receptors exist in cell membranes. The receptor protein is a homodimer of α and β subunits. Similar to other membrane receptor proteins, the insulin receptor has polarity, the α-subunits are extracellular, and the β-subunits are intracellular; see Figure 1. The hormone insulin, as a first messenger, triggers a cascade of reactions when it attaches to the α-subunit of the receptor. The β-subunit is then activated. The β-subunits of the receptor display tyrosine kinase enzyme activity; therefore, a sort of auto-phosphorylation occurs in this subunit of the protein. The consequence of this initial activation is the further phosphorylation of insulin receptor substrates; the one demonstrated in diagram 2 is IRS-1. This phosphorylation process in turn activates a cascade that results in the activation of other kinases and transcription factors that mediate the intracellular effects of insulin [60]. The consequence of this is the expression and insertion of GLUT4 receptor subtypes into the membranes of myocytes and lipocytes, as well as the biosynthesis of glycogen in the liver and skeletal muscle tissues. This biosynthetic process also converts glucose residues into fat (triglycerides) in the liver, adipose tissues, and lactating tissues of mammary glands. Indeed, all of these metabolic processes operate via the activation of phosphoinositol 3-kinase (PI_3_K) by insulin receptor substrate 1 (IRS-1). PI_3_K is a membrane-associated enzyme that catalyzes the conversion of phosphatidylinositol 4,5-bisphosphate (PIP_2_) into phosphatidylinositol 3,4,5-triphosphate (PIP_3_). This membrane phospholipid metabolite in turn activates another kinase known as protein kinase B (PKB), also called Akt or PKB/Akt. Once this is activated, PKB mediates the translocation of these vesicles (endosomes) that store GLUT4 in the cell membrane. These vesicles fuse with the membrane and deliver the GLUT4 proteins they contain to the cell membrane. This event increases the number of GLUT4 transporters in these membranes. PKB also inhibits glycogen synthase kinase (GSK) by another step of phosphorylation [61]. In other words, the substrate of GSK, glycogen synthase (GS), cannot be phosphorylated and thus remains active. This active enzyme acts as a regulatory point to limit the synthesis of glycogen from glucose residues. A series of similar dephosphorylation effects of GS (glycogen synthase) indirectly control the rate of glycolysis and lead to lipogenesis (in tissues that can do so), and controls the rate of gluconeogenesis in hepatic tissue. These dephosphorylation reactions stimulate the biosynthesis of fat and glycogen from glucose. As a consequence, glycogenolysis and gluconeogenesis by the liver are inhibited. Moreover, the hydrolysis of triglycerides into free fatty acids and glycerol in the lipocytes is also inhibited [62]. In the basal state of the cell, after leaving the Golgi network, newly synthesized GLUT4 proteins are stored in the insulin-responsive storage compartment, abbreviated as IRC [63]. The signal initiated by the binding of insulin to its receptor ends (briefly demonstrated in Figure 1) by the endocytosis and degradation of these two bound proteins (insulin and its receptor) [64]. The two main sites for this clearance are the liver and the kidneys. In hepatic tissue, the clearance occurs during the first passage through the liver and the kidneys clear insulin from the systemic circulation. The half-life of insulin from being released from the beta pancreatic cell up to degradation is about 4–6 min [65].

### 2.7. Physiological Effects of Insulin

The main effects are demonstrated in Table 2 [66,67,68,69,70,71,72,73]. Individuals with poorly controlled diabetes and undetected diabetes cases can encounter cognition problems attributed to cerebrovascular incompliance. In the brain, insulin enhances learning and verbal memory [74]. Cerebral insulin enhances thermo and glucoregulatory responses to food intake; this phenomenon points to the fact that cerebral insulin coordinates a wide spectrum of homeostatic processes in our body [75]. Further, insulin favors fertility through its impact on the hypothalamus via its effect on the secretion of gonadotropin-releasing hormone [76]. Glucose is the primary fuel oxidized by neurons (in non-starving states) to biosynthesize adenosine triphosphate (ATP) to ensure the continuance of the wide spectrum of neurons’ metabolic machinery. Further, it is the only fuel molecule relied on by human RBCs to synthesize ATP [77]. Insulin is needed to drive glucose molecules into the cytosol of cells to synthesize ATP. ATP (the energy currency of biological systems) is necessary for different phosphorylation processes that usually activate kinase types of enzymes or keep certain proteins inactive, which is mentioned elsewhere in this work. The Embden–Meyerhof–Parnas glycolytic pathway (an anaerobic metabolic process) yields two ATP molecules for each glucose molecule. This process also produces a substrate (pyruvate molecule) that enters the mitochondria to produce far more ATP in an aerobic process through the Szent–Györgyi–Krebs cycle in cooperation with the electron transport chain.

### 2.8. Treatment

In the early stages of IR, it is possible to hamper/stop the progression of the pathology. Oral hypoglycemic agents are the mainstay of therapeutic treatment in IR. These agents generally exert their actions via three main means: (1) increase the amount of insulin released by the pancreas, (2) increase the sensitivity of insulin receptors in different target organs in such a way that they harmonize optimally with GLUTs, (3) decrease the rate of glucose being absorbed from the intestine, and (4) help the body dispose of excess glucose through urination. Among these agents, metformin (Glucophage) is usually the first-line drug. It is prescribed at the initial stage of diagnosis, in addition to bodily exercise and weight loss strategies that patients are usually advised to perform. These agents also have their drawbacks, such as lactic acidosis; the safest among them is probably metformin [78]. Metformin decreases both gluconeogenesis and glucose absorption via the gastrointestinal tract; moreover, it increases the body’s sensitivity to insulin [79]. The details of these agents fall outside the scope of this review and thus will not be discussed.

In addition to oral hypoglycemic drugs, recent research has shown that IR can also be combatted by methods that control the amount of insulin needed by the body both by production de novo or when therapeutically taken by injection via insulin pumps [80,81]. A reduction in IR can be achieved by following low-carbohydrate and ketogenic diets. Individuals with IR are advised to consume fiber-containing food with a low amount of calories. The aim is to treat the obesity in these individuals if they should be so affected [82]. It is believed that fruit with high amounts of protein and fibers that also have low levels of calories should be recommended for this population, as these also contain antioxidants such as vitamins C, A, and E [83]. Many types of fruit such as avocado and bananas also contain considerable amounts of potassium, which is a necessary contributor to the control of blood pressure (hypertension). Many diabetic individuals also suffer from hypertension as a prominent milestone in the spectrum of metabolic syndrome. Daily exercise (bodily motion) is a preventive measure and an essential pillar of treatment to fight obesity and thus, indirectly, IR.

## 3. Discussion

The dilemma of IR cannot be explained as being simply dependent on obesity, as many prediabetes individuals in certain societies are skinny or have lean bodies [84]. There exist other plausible theories that have been proposed to elucidate the pathophysiology of IR. These are discussed in the following sections.

### 3.1. Lipotoxicity and Effects of Ceramide

The term lipotoxicity was first used by Unger et al. when they described the inhibition of the function of pancreatic beta cells to release insulin into circulation because of lipid overload in the body [85]. Not only this, but also an overload in the circulation of free fatty acids such as palmitate (16-carbon saturated fatty acid), activate CD36 or free fatty acid receptors (FFARs)—a process that causes cell stress and the formation of ceramides, lipid droplet, endoplasmic reticulum stress, mitochondrial dysfunction, and autophagy. These responses altogether contribute to beta cell damage and thus impaired insulin secretion into circulation [86]. Elevated plasma levels of free fatty acids, and thus fatty acid CoA (fatty acyl CoAs), are mostly observed in obese individuals, and cause inhibition of insulin-stimulated glucose uptake and thus glycogen synthesis [87]. Briefly, lipotoxicity is the state of accumulation of excess fat, which has a negative influence on glucose metabolism, in such a way that it causes derangement in several metabolic pathways in adipose tissue and different peripheral tissues such as the liver, heart, pancreas, and skeletal mycoses [88]. What makes the situation worse is the accumulation of lipid outside the lipocytes, or so called ectopic cells. According to results of many studies, one of these ectopic regions is the skeletal muscle [89]. These studies associate this abnormal fat deposition in the skeletal myocytes with insulin resistance and T2D. On the other hand, most other studies go along with the assertion that it is not the intramuscular deposited fat per se, but rather the intermediate lipid species such as ceramides, diacylglycerols (DAGs), and fatty acyl-CoAs that are the real cause of insulin resistance. Human studies indicate a connection between ceramides and insulin resistance. The accumulation of ceramides in the tissues occurs via the excessive supply of fatty acids—these could be saturated or unsaturated fatty acids. This accumulation process is most likely attributed to sphingolipid salvage or recycling of the pathway activity [90]. Increased diacylglycerols (DAGs) also contribute to the development of IR; DAGs translocate a protein kinase known as protein kinase Cε to the membrane, which in turn phosphorylates the insulin receptor and impairs its activity [91].

In other words, localization, composition, and the turnover of these intermediates together contribute to the pathophysiology of insulin resistance and, subsequently, T2D [92]. This assertion aligns with what is known as the adipose tissue expandability hypothesis of insulin resistance and metabolic syndrome [93]. The circulation of increased amounts of lipids influences both fatty acid utilization and intracellular signaling. This abnormal phenomenon has been related to insulin resistance in skeletal muscle and liver. Excess circulating fat is a burden on mitochondria, as these are the primary site for fatty oxidation to extract energy, that is, ATP (adenosine triphosphate) biosynthesis. As a byproduct, this process generates reactive oxygen species, which cause endoplasmic reticulum stress. These two factors hand-in-hand play a pivotal role in the pathophysiology of insulin resistance, a situation encountered especially in individuals with non-alcoholic fatty liver disease [94]. Another candidate from lipid species is the sphingolipid ceramide and diacylglycerols, as mentioned earlier. These metabolites have been shown to exhibit toxic effects on many classes of human cells such as pancreatic beta cells, liver, skeletal muscles, and cardiomyocytes [95]. The destructive effects of these lipid species on different cell types, and thus tissues, have been shown to be associated with metabolic syndrome, as documented by many considerable studies [90].

### 3.2. The Inflammatory and Autoimmunity Theory

The link between IR and inflammation goes back several decades. High doses of acetylsalicylic acid (aspirin), a NSAID (nonsteroidal anti-inflammatory drug), were found to reduce glucose levels in diabetic patients. In this aspect, corticosteroids have also been shown to have a therapeutic effect on the treatment of antibody-induced IR with eosinophilia according to a case report from Japan [96]. This fact goes hand-in-hand with the discovery of the role of tissue macrophages, which led us to change our vision about IR, T2D, obesity, and ultimately metabolic syndrome and explain that these are dependent on the autoimmune inflammation theory [97]. The accumulation of pro-inflammatory macrophages in adipose tissues of obese type 2 diabetics is prominent evidence of the relation between inflammation and IR [98], although the nature of the causal relationship in unclear, as it is not understood whether IR per se is the result of an inflammatory process, or vice versa [99]. It is well known that injury and infection activate the immune system in the human body. Provoking this system leads to a wide spectrum of destructive mechanisms against the infective organism and repairs the damaged tissue in the meantime. Tumor necrosis factor-α (TNF-α) is involved in the immune process that fights infection. This protein is an inflammatory parameter, which confirms the fact that IR is an inflammatory event [100]. The autoimmune factor has also been postulated as a predisposing element to IR. One of the known criteria of autoimmune pathogenesis is vitamin D deficiency. The deficiency of this essential fat-soluble micronutrient has been associated with a wide spectrum of autoimmune morbidities including IR; moreover, complications of diabetes may be aggravated by the deficiency of this vitamin [101]. Insulin resistance and the subsequent obesity is a sort of subtle chronic inflammation that impedes the action of insulin and hampers the release of this hormone. The recruitment of monocytes and the release of different chemokines and cytokines from different leukocyte subclasses suggest an innate immune response. According to some researchers, a response can also evolve into an autoimmune condition. Basically, both of these postulations back the inflammatory hypothesis of IR [102]. There exists a multitude of evidence in the medical literature to suggest that the autoimmune background of IR can coexist even with organ-specific autoimmune conditions, such as autoimmune thyroiditis [103].

### 3.3. Impact of Eosinophilia

Eosinophils are allergic pro-inflammatory leukocytes; once these are found in human adipose tissue, they dysregulate metabolism in the whole body and cause IR [96]. Infiltration of eosinophils into adipose tissue of obese individuals has been linked to metabolic disorders, such as IR to inflammation [104].

### 3.4. Oxidative Stress

Oxidative stress has also been taken into consideration as a potential predisposing factor to IR. In diabetes, an uncontrolled influx of free fatty acids and glucose into the cells is associated with an increase in mitochondrial machinery, and thus release of reactive oxygen species (ROS). The outcome of this process is an increase in oxidative stress. This process activates many other cellular-stress-sensitive pathways, which in turn hampers different cellular signaling pathways. Hydrogen peroxide (H_2_O_2_) is an element of ROS. This harmful metabolite, even in micromolar concentrations, can activate different stress kinases including c-Jun N-terminal kinase, IκB kinase, p38, and extracellular receptor kinase 1/2 (ERK 1/2). In turning down, this activation regulates the cellular responses to insulin, a process that impedes glucose uptake and thus inhibits glycogen and protein synthesis. Extensive exposure to ROS interferes with the transcription of GLUT4 and its translocation to the plasma membrane. A misbalance in the proportion of GLUT4 to GLUT1 will ensue, meaning that the GLUT4 level decreases against GLUT1 [105]. It is obvious from what has been mentioned so far that IR is a multifactorial morbidity, and that oxidative stress is among these. This condition occurs when the number of reactive species exceeds the abundancy of antioxidants. The overabundance of oxidative species is mechanistically connected to different tissues of our body, primarily skeletal muscle tissue. Skeletal muscles rely mainly on glucose as it is a fuel molecule for their function and, to a lesser extent, on fats. Free radicals, ROS, are the byproducts of different metabolic activities that occur in mitochondria. There are two mechanisms to explain the impact of ROS on IR, as follows: (1) the overproduction of H**_2_**O**_2_** and superoxide (O**_2_^−^**) ions in the aerobic situation occurs where oxygen (O**_2_**) is abundant, in other words, when ATP is in surplus; (2) the enhanced activation of NADPH oxidase (via angiotensin type II receptors) in skeletal myocytes generates H**_2_**O**_2_** and increases oxidative stress even further. NADPH is a potent reducing agent in eukaryotic cells. Recent studies support this concept, as the exposure of myocytes to oxidative stress stimulates serine kinase p38 mitogen-activated protein kinase (p38 MAPK). This protein in turn negatively affects insulin-dependent glucose transport activity, which is the flux of glucose molecules into skeletal myocytes [106]. These facts basically provide backing to the statement that having a lean body through regular exercise and consuming healthy foods that are rich in micronutrients (minerals and vitamins) that have antioxidant capacity, such as vitamins C, A, and E, as well as minerals such as selenium (available in tuna fish) and acids such as α-lipoic acid and essential polyunsaturated fatty acids (PUFAs) of omega-3 and omega-6, are important environmental factors to combat IR and thus T2D [16,107,108]. Animal studies have also revealed that mitochondrial oxidants impair insulin regulated GLUT4 translocation and thus contribute significantly to IR [109,110]. As has been discussed so far, one can realize that IR in addition to inevitable development of diabetes and its complications (if neglected) can cause a form of mild dementia and cognitive impairment, as a result of neurodegeneration. The reason behind these neurological complications is the neuronal oxidative damage occurring as a sequelae to the imbalanced redox state of neurons [111]. Insulin resistance causes a state of chronic hyperinsulinemia as a response to long-standing hyperglycemia, dyslipidemia, and a prolonged subtle pan-inflammation in all tissues of the body.

### 3.5. Mitochondrial Dysfunction

Dysfunction of these intracellular organelles emerges as one of the major etiological factors to many classes of morbidities in humans, among these IR. The major fuel species that mitochondria rely on to biosynthesize the energy currency, ATP, are glucose and fatty acids. This means that, when the oxidation of these molecules is inefficient, the ratio of ATP/O_2_ consumption is lower than what is needed. This results in an increased production of super oxide ions (O**_2_^−^**). This reactive oxygen radical increases the possibility of mutagenesis and stimulation of certain pro-inflammatory processes. In order to function properly, mitochondria need thiamin (a vitamin B sub-class in the vitamin B complex); this water-soluble micronutrient acts as a cofactor for essential enzymes that mitochondria must have for their optimal functioning, including transketolase, pyruvate dehydrogenase, and α-ketoglutarate [112]. In addition to these, other natural factors such as genetics and aging, which reduce the biogenetic capacity of mitochondria, play a role. Aging per se causes a reduction in insulin-stimulated glucose metabolism in skeletal muscles, which in turn increases fat accumulation in the liver, besides a reduction in mitochondrial oxidative phosphorylation by almost 40% [113]. All of these factors work together and contribute to IR in tissues targeted by insulin, such as skeletal muscles, adipose tissue, liver, and to a lesser degree the myocardium. Insulin resistance that arises from mitochondrial dysfunction may contribute to metabolic and cardiovascular morbidities. Moreover, IR is also improved when mitochondrial function improves [114]. Recent studies in healthy lean, elderly individuals and their healthy lean insulin-resistant offspring who developed T2D later have demonstrated the accumulation of lipid in their skeletal myocytes and suffering from IR. This metabolic defect was attributed to mitochondrial dysfunction and a reduction in mitochondrial density [115]. From what has been mentioned so far, one can observe the tight link between IR and mitochondrial dysfunction, especially with regard to the mitochondrial machinery, biosynthesis, and bioenergetics [116]. Renin-angiotensin stimulation and vitamin D deficiency are direct factors to release high levels of ROS, which is a risk factor in hypertension. Moreover, excess ROS formed in mitochondria cause apoptosis and degradation of mitochondrial DNA [117]. High levels of ROS are also a primary cause in the development of IR, as has been mentioned before. In the medical literature, one can find dozens of documents to pinpoint the link between mitochondrial dysfunction and insulin resistance [118,119].

Whether IR is the cause of mitochondrial dysfunction, or vice versa, and whether mitochondria are the main target for IR treatment is a subject of endless debate [120].

### 3.6. Impact of Platelets

The platelet count is an important indicator to probe different categories of morbidities that affect humans, and IR is one of these. In a study performed by Park et al. on Korean adolescents, it was concluded that the platelet count was positively linked to IR; moreover, the authors also state that platelet count could especially be a useful tool to identify adolescent IR [121]. Independent of the subtle low-grade inflammation that accompanies IR, IR per se in obese women contributes to platelet activation [122]. This is an observation that plausibly has something to do with female hormones. Moreover, the condition of activated platelets in obese women is linked to oxidative stress and inflammation, a condition that can be reversed to a certain extent with a well-designed weight loss program [123]. It an accepted fact that the platelet count is independently associated with IR in non-obese type 2 diabetic individuals [124]. This assertion elucidates the reality that platelet hyperactivity is multifactorial and cannot be explained only by a dependence on the association of obesity and IR. The reason for the increase in platelet count in IR is a subject of hot debate. Hyperglycemia promotes the glycation (non-enzymatic glycosylation) of platelet proteins and increases platelet reactivity. Indeed, both IR and deficiency increase this reactivity. Under physiological conditions, this peptide hormone (insulin) antagonizes platelet activation (thrombus formation). In other words, the deficiency of insulin relatively or absolutely will increase the reactivity of these enucleated tiny cells. As has been stated elsewhere in this work, diabetes mellitus is strongly associated with oxidative stress and inflammation. Microangiopathy (endothelial dysfunction), the pathognomonic complication of diabetes, promotes the activation of platelets passing through these narrow vessels by increasing the release of nitric oxide (NO) from endothelial cells of capillary vessels. Oxidative stress intensifies the biological action of NO, and thus promotes platelet activation [125].

### 3.7. Microbiota and Insulin Resistance

Obesity is a metabolic disease, caused by an intermingling of genetic, metabolic, and environmental factors. New evidence points to a role of gut microbiota in mediating the interaction of the host (patient) and the environment via extracting energy from the host (individuals) food that is otherwise indigestible by the host him/herself. This energy extraction releases different metabolites and cytokines as byproducts that affect the whole metabolic profile of the host. Normally, there exists a symbiotic relationship between the gut microbiota and the host. A microbial disbalance (dysbiosis) has been shown to arise in certain metabolic morbidities such as obesity [98]. The gut’s microbial composition can be affected by antibiotics, NSAIDs, and xenobiotics (components of the diet) that promote dysbiosis. The gut microbiome dysbiosis may change the intestinal barrier function and other different signaling pathways in the host. These changes are directly or indirectly linked to IR, and thus T2D. Any change in gut microbiota can cause the metabolic balance to deviate towards energy harvesting in the presence of diabetes and obesity. One should admit that the exact mechanism(s) behind the dynamics exerted by gut microbes and the impact of these dynamics on the host have not been elucidated [126]. Yet, there exist documents that demonstrate that microbiota suppress the expression of a protein known as fasting-induced adipose factor or “lipoprotein lipase inhibitor” (LPLi). This suppression causes microbiotas’ colonization, which enhances lipoprotein lipase (LPLase) activity, which in turn increases the storage of triglycerides driven from the liver [127]. Dysbiosis of gut microbiota and the metabolites they produce possibly initiates a subtle inflammatory reaction that can cause IR in obesity and T2D. One aspect that must be mentioned in this context is that there exists strong evidence that the “trio” interplay of microbiota, host immune machinery, and metabolism is a crucial participant in the pathophysiology of IR, the consequent obesity, and T2D [128,129]. The determination of the functional and compositional changes in microbiota and the relevant species of these that influence metabolic health, and thus leanness, is a challenging issue. The diet is certainly a major parameter in shaping the obesity-related dysbiosis. Adding to this, it is important to elucidate dietary components, and dietary patterns also have a lot to confer on healthy gut microbiota of the host. As a matter of fact, how microbiota affect human health and disease is a hot area of investigation [130]. The link between the dysbiosis of the gut microbiome, the host metabolic profile, and the development of IR and its subsequent complications provides hope for finding viable solutions to treat this endemic morbidity, namely IR.

### 3.8. Drug-Induced Insulin Resistance

This issue has been a great challenge for drug designers for more than a century. As we know there is a fine line of balance between the therapeutic effects of the majority of drug classes used to treat human morbidities and their side effects, that is, between the pharmacodynamics and pharmacokinetics of these agents. Among these pharmacodynamic issues are the drawbacks of each drug. IR is one of these morbidities, and a discussion of all of the drug categories and the mechanisms they exhibit when causing a side effect falls outside the scope of this review work; one can see this issue in more detail in [131]. Many psychotropics (antipsychotics, antidepressants, and anxiolytics) exert this drawback even when used in therapeutic doses; in most cases, the mechanism of this undesired effect is not well understood, especially at neuronal levels. Antipsychotics, both first- (typical antipsychotics) and almost all second-generation agents (atypical antipsychotics), can cause this side effect to a considerable extent. This is attributed to the exact mechanism of how these drugs operate at the cellular level, which is not clear. Moreover, these therapeutic agents do not have a selective action, and thus affect tremendous classes of neuronal membrane proteins (receptors, membrane-associated enzymes) at both the cellular and intracellular organelle levels, especially the mitochondria. Mitochondrial dysfunction per se has been considered as a causative factor for the development of both psychosis and IR, as has been demonstrated elsewhere in this review, thus these agents can further worsen the morbid situation. IR occurs in schizophrenia with or without the administration of antipsychotic agents [132]; this is attributed to the mitochondrial dysfunction that coexists or is itself a causative factor of psychosis [133,134]. The impotency and non-selective therapeutic action of first-generation antipsychotic agents has encouraged drug designers to make more selective and potent agents that, at the same time, exert the minimum possible side effects, including neuroleptic malignant syndrome. These agents are known now as second-generation (atypical and/or nonconventional) antipsychotics [135]. Unfortunately, it turned out later that these new agents cause unwanted metabolic effects, among which is IR, with the worst agent among these in this context (so far known) being olanzapine [136]. What makes the issue worse is that some of these new agents also release ROS, which directly affects mitochondrial function [137]. The majority of these agents exert their therapeutic effects via antagonizing dopamine receptors and especially the subclass D_2_ [138]. How these agents predispose to IR is a matter of speculation, but there exist plausible concepts to explain these mechanisms; namely, (1) it seems that metabolic changes associated with the use of second-generation antipsychotics occur independently of weight gain—these changes increase the feeling of hunger and thus food intake. Taken together, this suggests that these agents exert a sort of direct effect on tissues of the human body independent of mechanisms that regulate the eating pattern of psychotic individuals [139]. Other mechanisms that have been postulated include the following: (2) antipsychotics inhibit insulin signaling pathways in target cells such as skeletal myocytes, hepatocytes, and adipocytes, thus causing IR in these tissues; (3) atypical antipsychotics cause obesity that can result in hyperlipidemia of the free fatty acid type (lipotoxicity) that can predispose inflammation, which further causes IR; and (4) atypical agents might cause direct damage to β-cells of the pancreas, leading to apoptosis and thus dysfunctioning of these endocrine cells [140]. The recent trends in explaining the pathophysiology of diabetes necessitate the coexistence of β-cell damage and IR, a dilemma to understand whether IR is the cause of diabetes or vice versa.

### 3.9. Membrane Function and Membrane Pacemaker Hypothesis

This theory hypothesizes that variation in basal metabolic rate (BMR) among vertebrates, including humans, is controlled by the physical characteristics of cell membranes [141,142]. The concept of this hypothesis was built upon the observation that certain biological processes in eukaryotic cells demand energy to maintain their activity. An example of this is the activity of the “Na^+^/K^+^ ATPase pump”. By hydrolyzing ATP, this protein maintains the homeostatic states of these two cations (Na^+^ and K^+^) in a balance between intra and extracellular compartments [143]. To propagate such membranal activities, the fatty acid composition of membranal phospholipids plays a large role, as this is directly correlated with membrane integrity and fluidity. The fluidity of biomembranes (and those of intracellular organelles, especially mitochondria) is controlled by two important factors, namely (1) the fatty acid contents of the membranal glycerophospholipids, where the shorter and unsaturated nature of these favors fluidity; and (2) the membranal cholesterol content is important. The latter in fact is a modulator and thus controls the fluidity-rigidity state of these membranes. Membranal integrity is an essential demand for the optimal functioning of membrane proteins (receptors, transporters, pumps, and membrane-associated enzymes). The integrity of membranes also influences the arrangement of the alignment of membrane proteins and makes substrates available for different membrane-associated enzymes. Receptors that are not aligned in an optimal position within the membrane cannot mediate the transduced hormonal signal, as they should. As membrane proteins, the concept is true for glucose transporters (GLUTs) to allow the influx of glucose molecules into the cells. The same is true for the membrane-associated enzymes, which have to reach the substrates in an orientation so that they can catalyze biological reactions [144]. There is a strong correlation between the metabolic rate of a cell and the polyunsaturation of its membrane phospholipids [145]. Most of the fatty acid content of these membranal phospholipids, especially those at the *sn-2* position, is of an essential fatty acid type, that is, ω-3 and ω-6 variants (these are essential biomolecules; they cannot be biosynthesized in human body). The abundancy of PUFAs in membrane glycerophospholipids is a diet-induced process and the ratio of ω-3/ω-6 also has a substantial impact on the diseases of metabolic syndrome and thus IR [142]. Moreover, ω-3 eicosapentaenoic acid (EPA) has a favorable effect on skeletal myocytes in switching these to glucose utilization rather than fatty acids as fuel molecules to synthesize ATP in fed states. This switching is a normal process in lean healthy individuals, but not in IR, obese, and type 2 diabetic individuals. People with these morbidities have a reduced capacity to oxidize lipids during fasting states [146]. Loss of this switching capability is known as metabolic inflexibility [147]. A reduced postprandial ability to switch from fatty acid oxidation to glycolysis to biosynthesize the energy currency, ATP, has been detected in individuals with IR (impaired glucose tolerance), which reflects that switching inflexibility plays a pivotal role in the development of T2D [148,149].

### 3.10. N-Glycosylation of Membrane Proteins

This is the glycosylation at the amino end of an asparagine residue of the membranal polypeptides (proteins). Accumulating evidence indicates that N-glycans play a major role in preventing IR by maintaining the GLUTs in a proper orientation (alignment); this phenomenon is also controlled by membrane fluidity (integrity), influenced by the PUFAs that exist in membranal glycerophospholipids, as mentioned earlier. Defective N-glycosylation of T cells (T lymphocytes) has been implicated in the pathogenesis of type I diabetes. Moreover, intensive research exists where N-glycan alterations have successfully been used as a probe to identify individuals with rare types of diabetes such as the HNF1A-MODY (Maturity Onset Diabetes of the Young), as well as to evaluate the functional significance of novel diabetes-associated mutations [150]. In addition to N-glycans, glycosyltransferases (proteins that catalyze the transfer of a sugar moiety from an activated donor sugar onto saccharide and nonsaccharide acceptors) have emerged as potential targets in the treatment of diabetes [151]. The significance of N-glycan is obvious in the stabilization and maturation of newly biosynthesized membrane transporter protein GLUT4 and their localization in the membrane in response to insulin treatment, whereas glycosylation mutant GLUT4 is deprived of this feature [152]. Glucose transporters are membrane proteins that are encoded by their distinct corresponding genes located in DNA polymers of the chromosomes. Mutations that might occur in these genes result in misfolded and abnormal membrane proteins. As is the case with other proteopathies, these membrane proteins (GLUTs) will not function soundly and thus cause hyperglycemia, which results in insulin resistance [153,154,155].

### 3.11. Vitamin D Deficiency and Insulin Resistance

As is the case with gestational diabetes, some evidence also exists to link other forms of IR to vitamin D deficiency. One of the major studies, involving 5677 subjects with impaired glucose tolerance, showed that vitamin D supplementation increased insulin sensitivity by 54% [156]. Other studies also found that increased vitamin D intake improves insulin sensitivity [157]. Vitamin D is a fat-soluble micronutrient that has a hormone-like action. It is not exactly clear how this essential metabolite reduces IR, but research on animals has demonstrated that it reduces IR, probably via its effect on calcium and phosphorus metabolism and upregulation of the insulin receptor gene [158]. According to a Canadian study, for individuals who are at a high risk of developing diabetes (prediabetes) or those with newly diagnosed type 2 diabetes, vitamin D supplementation for 6 months significantly increased their peripheral insulin sensitivity and β-cell function. This finding suggests that vitamin D may slow metabolic deterioration in this group of the population [159].

### 3.12. Influence of COVID-19

Pancreatitis of any etiological background is also expected to affect endocrine functions of this mesodermally originated digestive/endocrine system gland. It affects both the exocrine and endocrine functions of the major gland of the human body (pancreas). This includes both carcinomas and infections [160,161]. Recent research data show that COVID-19 triggers insulin resistance in patients; it causes chronic metabolic morbidities that were non-existent prior to infection, such as insulin resistance. It is believed that SARS-CoV-2 causes direct damage to their insulin-secreting pancreatic β-cells. It is worth mentioning that some patients may require insulin to start with as a therapeutic agent, and those who already have diabetes prior to COVID-19 may need their insulin doses to be increased [162]. It is clear from what has been demonstrated in this subtopic that COVID-19 causes insulin resistance and the opposite appears to be true—that is, those with diabetes have a greater liability of developing COVID-19.

The reason individuals with diabetes have a tendency to become infected with SARS-CoV-2 is explained plausibly as follows: COVID-19 decreases the expression of angiotensin-converting enzyme-II (ACE-II), a process that exaggerates the expression of angiotensin-II, which in turn provokes subsequent insulin resistance [163,164].

### 3.13. How to Combat Insulin Resistance?

It is possible to reverse IR, particularly in its initial stages. It is also true that we cannot do much with genetic factors that, in most cases, underlie and predispose this morbidity, but we can do a lot with the environmental factors that provoke this morbidity, which, if not controlled, will lead to a flare-up of the situation. Among these measures is the weight loss achieved by performing regular bodily exercise [165,166]. Sufficient water intake is an important measure to decrease hyperglycemia. We know that water does not contain calories, thus it is one of the best drinks diabetic individuals should consider and consume. There are many studies confirming this assertion about water [167], which contrasts that of alcohol beverages. Diabetic individuals should avoid heavy drinking, as this can cause ketoacidosis and hypertriglyceridemia. The worst thing for them is to engage in heavy drinking in the fasting state, as this can cause the opposite action—hypoglycemia, which increases the risk of death from non-cardiovascular causes in diabetic individuals [168]. Water indeed flushes out extra glucose from the blood [169,170]. Dehydration also causes an increase in the level of vasopressin, a hormone that is possibly a risk factor for the development of hyperglycemia [171]. Increased water intake may prevent or at least delay the onset hyperglycemic state and subsequently the onset of T2D [172]. According to climate and gender, normal water intake ranges from 1.6 to 2.0 liters per day (European Food Safety Authority). In these cases, it is necessary to change dietary habits; that is, for individuals to consume fruit and vegetables in order to obtain essential micronutrients (cannot be biosynthesized de novo). These include both water- and fat-soluble vitamins, many of which have antioxidant properties, e.g., vitamins C, A, and E. This is necessary for many reasons, including to combat the subtle inflammatory state that coexists with insulin resistance—a fact that is obvious even in animal model studies [173]. Moreover, heavy body exercise can also lead to the release of ROS; therefore, moderate intensive exercise is recommended as a starting point. Among other pieces of advice, these individuals are encouraged to give up smoking, as this habit also contributes to increasing the effects of ROS as a result of the cigarette smoke. Foods with high protein and fiber contents and low fat and carbohydrates contents (low glycemic index) are also important to supply the body with essential amino acids to build up the protein reservoir of the body. This is necessary for the biosynthesis of primary macromolecules of the body, including fundamental enzymes of the mitochondria. Properly functioning mitochondria is a key issue to solve the dilemma of IR. Thiamin supplements are necessary to improve mitochondrial function because the derivatives of this essential micronutrient work as coenzymes of many essential enzymes in mitochondria. The integrity of membranes and their proteins is pivotal for cellular metabolic function as well as the oxidative reaction of mitochondria. In this context, vitamin E is important as an antioxidant to protect biomembranes of our cells and associated organelles. Polyunsaturated fatty acids (PUFAs), the constituent hydrocarbon moieties of the glycerophospholipids, are also fundamental for the fluidity of these membranes and their modulation. This is necessary for the optimal functioning of these membranes. Getting 7–8 h of sleep is important to maintain the blood glucose at healthy levels. For better quality of sleep, caffeine and/or alcohol should be avoided late in the day. Peer support groups might also be of help. One can learn from the experiences of others, which is important, as is practiced in different countries of the globe. Metformin is a good therapeutic agent both to control blood glucose levels and to assist our bodies with getting rid of extra pounds of body fat. As aging per se is potentially a contributing factor to the development IR, therapy with this oral hypoglycemic agent has another advantage over hypoglycemic agents from other classes in that it has antiaging effects, giving its use a double benefit [174]. Endothelial dysfunction as a result of of microangiopathy in T2D individuals is a key factor for the development of cardiovascular morbidities. Therefore, preserving endothelial function is a potential target to prevent these complications in T2D individuals [175]. The state of hyperinsulinemia that occurs in insulin resistance and T2D increases the rate of anaerobic myocardial metabolism (it increases lactate extraction) and thus negatively affects the function of the left ventricular contractile, a plausible and possible etiological factor for the development of cardiomyopathy and thus heart failure [176].

## 4. Conclusions

Insulin resistance and its other face, namely T2D, have multifactorial etiology, which are genetic and environmental. It is true that we cannot do so much currently with the genetic factors, but we can do a lot with the environmental ones. A healthy diet with a low glycemic index and high antioxidant content, daily bodily motion to prevent weight gain and obesity, avoiding social stress, and giving up smoking are important measures to prevent the progression of insulin resistance to diabetes and most probably to eliminate insulin resistance as well. As a matter of fact, regular bodily exercise as a sort of change in lifestyle of individuals with T2D, without a therapeutic agent, can abort (improve) and/or reverse T2D. This is attributed to the contraction of skeletal myocytes, which enhances the translocation of GLUT4-containing vesicles to the cell membrane (sarcolemma)—that is to say, to open the gates for glucose molecules to be internalized in these cells. The significant advantage here is that the mechanism of this translocation is insulin-independent. Those individuals that at real risk of developing T2D (insulin-resistant subjects) can probably decrease this possibility by incorporating physical bodily exercises into their daily routine. This issue is especially vital for those who have sedentary professions.

It is, therefore, important to abort IR whenever possible in its initial stages. The problem with this morbidity is that one might not be able to notice any of the significant symptoms of the diseases. Therefore, an annual medical check-up is necessary, especially for overweight individuals in the age group above 40 years and especially those with a family history of diabetes.

## 5. Future Perspectives

Future directions in this research will be to design real studies to investigate IR in depth. Finding candidates for participation in such studies might be a difficult task, especially in countries where annual check-up routines do not exist for different reasons. As gestational diabetes is a variant of IR, women with gestational diabetes could be perfect candidates (participants) upon whom to conduct such research. Pregnant women usually undergo a thorough antenatal investigation in most countries. In this sense, it might be convenient to involve these women voluntarily in investigations of this type. Much more should be done to understand the real pathophysiology of IR and the hidden aspects of its etiology, and designing better and more selective hypoglycemic agents with fewer drawbacks to overcome prediabetes is an option. The currently available hypoglycemic agents have drawbacks, some of which can be serious and affect insulin-resistant individuals negatively. Pancreas transplantation has been an option, especially for type 1 diabetes (insulin-dependent diabetes). The currently available data in this context are very limited, especially in terms of the long-term metabolic run of such procedures and the technical surgical difficulties for such operations. Advances in immunosuppression have improved quality of life after transplantation as the newly transplanted pancreas could be rejected by the recipient individual. For T2D, stem cell transplant can reduce the metabolic symptoms of the disease and delay the progression of the inevitable complications of T2D in such a way that it might also improve the patient’s quality of life. Still, we have to admit that the procedure cannot offer a radical treatment to these diabetic individuals that will reduce its symptoms and metabolic complications.

## Figures and Tables

**Table 1:** The glycemic indices (GIs) of different food and nutrients.

Foodstuff and GI	Examples of Food
Food with a low GI range (55 or less)	Monosaccharides: fructose; tagatose. Pulses (beans): black; kidney; lentil; chickpea; pinto. Seeds (small): sesame; flax; sunflower; poppy; pumpkin; hemp. Nuts: walnuts; cashew; peanuts. Grains: wheat (durum, spelt, kamut); millet; oat; rye; rice; barley. Sweet fruits: peaches; strawberries; mangos. Vegetables: most vegetables; unpeeled sweet potatoes and mushrooms.
Food with medium GI ranges (56–69)	Table sugar; regular ice cream; cranberry juice; grape juice. Enriched whole wheat; basmati rice; unpeeled potatoes; peeled sweet potatoes; pita bread. Raisins; prunes; pumpernickel bread.
Food with high GI ranges (70 and above)	Sugars: glucose: dextrose; grape sugar; high fructose corn syrup; maltose; maltodextrins. White bread (from endosperm). Most white rice (from endosperm). Peeled potatoes Extruded breakfast cereals; corn flakes.

Based on [24,25,26,27].

**Table 2:** Direct and indirect metabolic effects of insulin.

Parameter	Physiological Function	Improper Function/Decrease in Insulin
Glucose	Stimulates glucose uptake via insertion of GLUT4 in the membranes of myocytes and lipocytes.	Increase in blood glucose concentration.
Triglycerols (fat)	Increases lipogenesis by forcing lipocytes to take in glucose.	Decrease in lipogenesis and hyperglycemia.
Fatty acids	Increased esterification to triglycerides (neutral lipids).	Lipolysis of triglycerides to fatty acids and glycerol.
Lipolysis	Decreases lipolysis and decreases free fatty acid and glycerol in the circulation.	Hyperlipidemia
Glycogen	Induces glycogen synthesis, by activation of the hexokinase that activates glucose by adding a phosphate, a process that traps glucose inside the cell.	Inhibits glycogen synthesis by reverse steps that induce glycogen synthesis.
	Inhibits glucose-6-phosphatase, which dephosphorylates glucose.	
	Activates both phosphofructokinase and glycogen synthase, which are responsible for glycogen synthesis.	
Gluconeogenesis and glycogenolysis	Decreases these two processes by decreasing glucose synthesis from noncarbohydrate biomolecules mainly in the liver.	Gluconeogenesis in the liver from diverse substrate biomolecules.
Protein	Decreases protein breakdown	Proteolysis is eased, as is the case in advanced cases of diabetes.
Autophagy	Deceleration of degradation of damaged organelles.	Autophagy is accelerated.
Arterial muscle tone	Increases this, especially arterioles and micro-arteries, and thus increases blood flow.	Reduces blood flow in these by allowing muscles to contract.
Gastric chlorhydria	Increases hydrochloric acid secretion by the gastric parietal cells.	T he occurrence of the reverse process is expected.
Potassium uptake	Forces glycogen-synthesizing cells to absorb potassium with water from the extracellular fluids via translocation of the Na^+^/K^+^-ATPase to the membranes of skeletal myocytes.	Inhibits potassium absorption.
Renal sodium excretion	Decreases excretion of renal sodium.	The reverse process occurs.

Based on [66,67,68,69,70,71,72,73], GLUT4: glucose transporter type 4, Na+/K+-ATPase: ATP dependent sodium/potassium pump.

## Data Availability

Not applicable.

## Abbreviations

Insulin resistance = IR; type 2 diabetes = T2D; reactive oxygen species = ROS; glucose transporter(s) = GLUTs; polyunsaturated fatty acids = PUFAs.
